# Supplementary material for: A novel angiotensin I-converting enzyme inhibitory peptide derived from the trypsin hydrolysates of salmon bone proteins
Source: PLoS One. 2021 Sep 2;16(9):e0256595. doi: 10.1371/journal.pone.0256595 (PMC8412326; doi:10.1371/journal.pone.0256595)
Supplement: S1 Fig — The predicted DH and the desirability value for optimum selected conditions of different independent variables for hydrolysis, as shown in (a). The predicted ACE-inhibitory activity (IC50; μg/mL) and the desirability value for optimum selected conditions of different independent variables for hydrolysis, as shown in (b). (DOCX) [file pone.0256595.s001.docx]

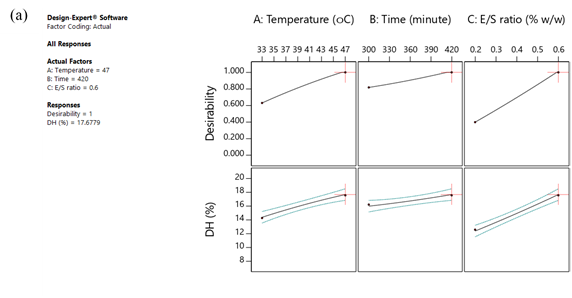


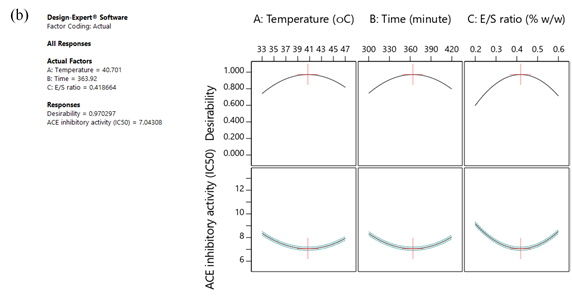


**S1 Fig.** The predicted DH and the desirability value for optimum selected conditions of different independent variables for hydrolysis, as shown in (a). The predicted ACE-inhibitory activity (IC_50_; µg/mL) and the desirability value for optimum selected conditions of different independent variables for hydrolysis, as shown in (b)
