# Supplementary material for: A novel angiotensin I-converting enzyme inhibitory peptide derived from the trypsin hydrolysates of salmon bone proteins
Source: PLoS One. 2021 Sep 2;16(9):e0256595. doi: 10.1371/journal.pone.0256595 (PMC8412326; doi:10.1371/journal.pone.0256595)
Supplement: S2 Fig — (PDF) [file pone.0256595.s002.pdf]

Spectrum Analysis Report

Date: 03/05/2020 Time: 12:41

FileName: C:\Users\onrapak.r\Desktop\John 4Mar2020\F7 Salmon_GE1_01_19932.mgf

Sequence Name:	Peptide	Formula:		Parentmass:	1070.599	Mass Error:	-0.931	MH+ (mono):	1071.530	MH+ (avg):	1072.261
Tolerance (Da):	0.500	Number of Peaks:	19	Above Threshold:	19	Assigned Peaks:	11	Not assigned Peaks:	8	Threshold (a.i.):	0.000

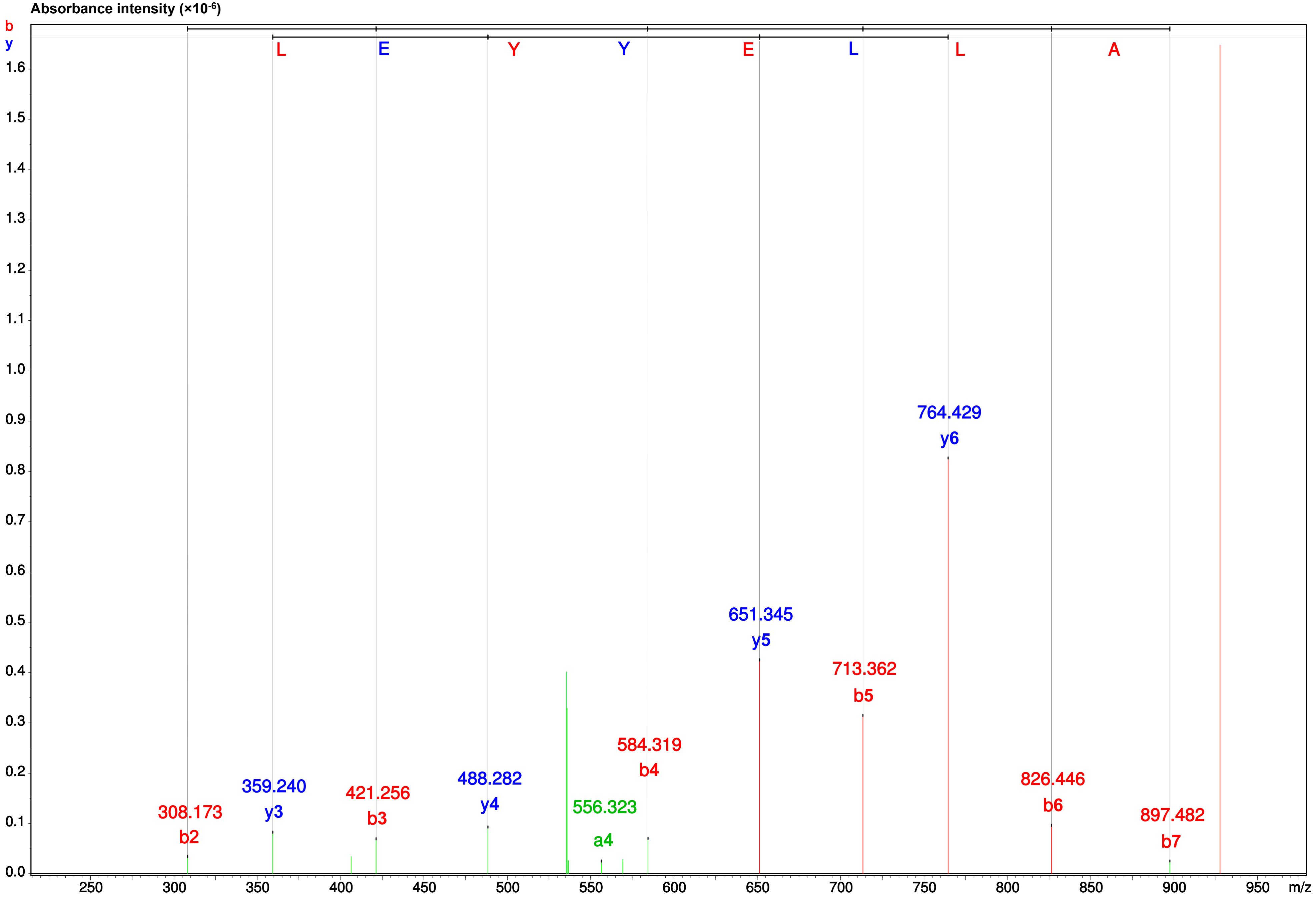

Spectrum Analysis Report

Date: 03/05/2020 Time: 12:41

FileName: C:\Users\onrapak.r\Desktop\John 4Mar2020\F7 Salmon_GE1_01_19932.mgf

Sequence data:

Peptide

Intensity Coverage:	100.0 % (3446302 cnts)	Sequence Coverage MS:	0.0%
Sequence Coverage MS/MS:	0.0%	pI (isoelectric point):	0.0

Display Parameter:

Sequence Name:	Peptide	Parentmass:	1070.599	Mass Error:	-0.931	MH+ (mono):	1071.530	MH+ (avg):	1072.261	Threshold (a.i.):	0.000	Tolerance (Da):	0.500
Number of Peaks:	19	Above Threshold:	19	Assigned Peaks:	11	Not assigned Peaks:	8						

Peaklist:

Peak	Mass	Intensity	Peak	Mass	Intensity	Peak	Mass	Intensity	Peak	Mass	Intensity	Peak	Mass	Intensity	Peak	Mass	Intensity	Peak	Mass	Intensity	Peak	Mass	Intensity
1	0.000	0.000	2	1.007	1258.000	3	308.173	36629.000	4	359.240	85118.000	5	406.196	34012.000	6	421.256	71873.000	7	488.282	95021.000	8	535.301	401652.000
9	535.803	328778.000	10	536.553	25966.000	11	556.323	27734.000	12	569.258	28468.000	13	584.319	72776.000	14	651.345	427659.000	15	713.362	317290.000	16	764.429	828988.000
17	826.446	98581.000	18	897.482	27422.000	19	927.493	1647350.000															

Calculated Masses:

FCLYELAR 2: Carbamidomethyl (C) Peptide

N-Term.	Ion	a	b	y	C-Term.	Ion
1	F	120.081	148.076	175.119	8	R
2	C	280.111	**308.106**	246.156	7	A
3	L	393.195	**421.190**	**359.240**	6	L
4	Y	**556.259**	**584.254**	**488.283**	5	E
5	E	685.301	**713.296**	**651.346**	4	Y
6	L	798.385	**826.380**	**764.430**	3	L
7	A	869.423	**897.418**	924.461	2	C*
8	R	1025.524	1053.519	1071.529	1	F

Copyright © 1999-2008 Bruker Daltonik GmbH
