# Supplementary material for: A novel angiotensin I-converting enzyme inhibitory peptide derived from the trypsin hydrolysates of salmon bone proteins
Source: PLoS One. 2021 Sep 2;16(9):e0256595. doi: 10.1371/journal.pone.0256595 (PMC8412326; doi:10.1371/journal.pone.0256595)
Supplement: S1 Table — (DOCX) [file pone.0256595.s003.docx]

**S1 Table.** Preliminary assessment

| **Temperature (°C)** | **DH (%)** | **ACE-inhibitory activity (IC_50_; µg/mL)** |
| --- | --- | --- |
| 30 | 13.9248 ± 0.8309 | 18.5100 ± 0.3536 |
| 40 | 19.7534 ± 0.7380 | 10.7300 ± 0.1273 |
| 50 | 19.1805 ± 0.7462 | 13.9000 ± 0.5798 |
| 60 | 17.4712 ± 0.2035 | 12.9250 ± 0.2758 |

| **Time (min)** | **DH (%)** | **ACE-inhibitory activity (IC_50_; µg/mL)** |
| --- | --- | --- |
| 120 | 11.4336 ± 0.1093 | 14.7000 ± 0.1093 |
| 180 | 12.3476 ± 0.9606 | 12.6050 ± 0.9606 |
| 240 | 12.0797 ± 0.0212 | 11.2200 ± 0.0212 |
| 300 | 12.5953 ± 0.0181 | 8.7495 ± 0.0181 |
| 360 | 13.8535 ± 0.0591 | 7.1080 ± 0.0591 |

| **E/S ratio (% w/w)** | **DH (%)** | **ACE-inhibitory activity (IC_50_; µg/mL)** |
| --- | --- | --- |
| 0.1 | 7.1226 ± 0.3719 | 12.2600 ± 0.5515 |
| 0.2 | 8.2080 ± 0.0247 | 11.9450 ± 0.1768 |
| 0.4 | 10.8194 ± 0.2795 | 8.1990 ± 0.4200 |
| 0.6 | 12.9087 ± 0.1146 | 9.0965 ± 0.1435 |
| 0.8 | 14.1133 ± 0.2381 | 11.5900 ± 0.6364 |
