# Supplementary material for: A novel angiotensin I-converting enzyme inhibitory peptide derived from the trypsin hydrolysates of salmon bone proteins
Source: PLoS One. 2021 Sep 2;16(9):e0256595. doi: 10.1371/journal.pone.0256595 (PMC8412326; doi:10.1371/journal.pone.0256595)
Supplement: S2 Table — (DOCX) [file pone.0256595.s004.docx]

| **No.** | **Run** | **Space type** | **Factor 1** | **Factor 2** | **Factor 3** | **%DH** | **%DH** | **Error** |
| --- | --- | --- | --- | --- | --- | --- | --- | --- |
|  |  |  | **A : Temperature (℃)** | **B : Time (min)** | **C : E/S ratio (% w/w)** | **(Actual)** | **(Predicted)** |  |
| 1 | 4 | Factorial | 33 | 300 | 0.2 | 9.35 | 9.02 | 3.63 |
| 2 | 18 | Factorial | 47 | 300 | 0.2 | 11.88 | 11.56 | 2.75 |
| 3 | 16 | Factorial | 33 | 420 | 0.2 | 9.60 | 9.62 | -0.24 |
| 4 | 1 | Factorial | 47 | 420 | 0.2 | 12.57 | 12.37 | 1.63 |
| 5 | 3 | Factorial | 33 | 300 | 0.6 | 12.90 | 12.89 | 0.08 |
| 6 | 5 | Factorial | 47 | 300 | 0.6 | 16.22 | 15.99 | 1.46 |
| 7 | 14 | Factorial | 33 | 420 | 0.6 | 14.26 | 14.37 | -0.74 |
| 8 | 10 | Factorial | 47 | 420 | 0.6 | 17.56 | 17.68 | -0.66 |
| 9 | 15 | Axial | 28.24 | 360 | 0.4 | 10.00 | 10.03 | -0.21 |
| 10 | 6 | Axial | 51.76 | 360 | 0.4 | 14.66 | 14.94 | -1.86 |
| 11 | 9 | Axial | 40 | 259.2 | 0.4 | 11.77 | 12.19 | -3.50 |
| 12 | 12 | Axial | 40 | 460.8 | 0.4 | 14.25 | 14.12 | 0.91 |
| 13 | 19 | Axial | 40 | 360 | 0.064 | 8.92 | 9.31 | -4.17 |
| 14 | 11 | Axial | 40 | 360 | 0.736 | 17.11 | 17.02 | 0.53 |
| 15 | 17 | Center | 40 | 360 | 0.4 | 13.54 | 12.91 | 4.89 |
| 16 | 20 | Center | 40 | 360 | 0.4 | 12.86 | 12.91 | -0.38 |
| 17 | 13 | Center | 40 | 360 | 0.4 | 12.42 | 12.91 | -3.77 |
| 18 | 7 | Center | 40 | 360 | 0.4 | 12.31 | 12.91 | -4.67 |
| 19 | 2 | Center | 40 | 360 | 0.4 | 12.95 | 12.91 | 0.33 |
| 20 | 8 | Center | 40 | 360 | 0.4 | 13.27 | 12.91 | 2.81 |

**S2 Table.** Experimental design matrix of CCD and corresponding results (degree of hydrolysis)
