# Supplementary material for: A novel angiotensin I-converting enzyme inhibitory peptide derived from the trypsin hydrolysates of salmon bone proteins
Source: PLoS One. 2021 Sep 2;16(9):e0256595. doi: 10.1371/journal.pone.0256595 (PMC8412326; doi:10.1371/journal.pone.0256595)
Supplement: S3 Table — (DOCX) [file pone.0256595.s005.docx]

**S3 Table.** Experimental design matrix of CCD and corresponding results (ACE-inhibitory activity)

| **No.** | **Run** | **Space type** | **Factor 1** | **Factor 2** | **Factor 3** | **IC_50_ (µg/mL)** | **IC_50_ (µg/mL)** | **Error** |
| --- | --- | --- | --- | --- | --- | --- | --- | --- |
|  |  |  | **A : Temperature (℃)** | **B : Time (min)** | **C : E/S ratio (% w/w)** | **(Actual)** | **(Predicted)** |  |
| 1 | 4 | Factorial | 33 | 300 | 0.2 | 12.14 | 12.01 | 1.11 |
| 2 | 18 | Factorial | 47 | 300 | 0.2 | 11.65 | 11.50 | 1.26 |
| 3 | 16 | Factorial | 33 | 420 | 0.2 | 11.78 | 11.78 | 0.01 |
| 4 | 1 | Factorial | 47 | 420 | 0.2 | 10.49 | 10.45 | 0.34 |
| 5 | 3 | Factorial | 33 | 300 | 0.6 | 10.44 | 10.45 | -0.08 |
| 6 | 5 | Factorial | 47 | 300 | 0.6 | 10.91 | 10.88 | 0.25 |
| 7 | 14 | Factorial | 33 | 420 | 0.6 | 10.82 | 10.94 | -1.08 |
| 8 | 10 | Factorial | 47 | 420 | 0.6 | 10.44 | 10.54 | -1.00 |
| 9 | 15 | Axial | 28.24 | 360 | 0.4 | 10.51 | 10.50 | 0.08 |
| 10 | 6 | Axial | 51.76 | 360 | 0.4 | 9.70 | 9.75 | -0.49 |
| 11 | 9 | Axial | 40 | 259.2 | 0.4 | 10.34 | 10.50 | -1.56 |
| 12 | 12 | Axial | 40 | 460.8 | 0.4 | 10.15 | 10.03 | 1.24 |
| 13 | 19 | Axial | 40 | 360 | 0.064 | 12.55 | 12.72 | -1.37 |
| 14 | 11 | Axial | 40 | 360 | 0.736 | 11.63 | 11.50 | 1.17 |
| 15 | 17 | Center | 40 | 360 | 0.4 | 7.26 | 7.08 | 2.66 |
| 16 | 20 | Center | 40 | 360 | 0.4 | 7.10 | 7.08 | 0.33 |
| 17 | 13 | Center | 40 | 360 | 0.4 | 7.12 | 7.08 | 0.55 |
| 18 | 7 | Center | 40 | 360 | 0.4 | 7.11 | 7.08 | 0.45 |
| 19 | 2 | Center | 40 | 360 | 0.4 | 6.87 | 7.08 | -2.85 |
| 20 | 8 | Center | 40 | 360 | 0.4 | 7.00 | 7.08 | -1.05 |
