# Supplementary material for: A novel angiotensin I-converting enzyme inhibitory peptide derived from the trypsin hydrolysates of salmon bone proteins
Source: PLoS One. 2021 Sep 2;16(9):e0256595. doi: 10.1371/journal.pone.0256595 (PMC8412326; doi:10.1371/journal.pone.0256595)
Supplement: S4 Table — (DOCX) [file pone.0256595.s006.docx]

**S4 Table.** Kinetic study

0 mM FCLYELAR (No inhibitor)

| **HHL (mM)** | **1/S** | **1/V** | | | **Average** |
| --- | --- | --- | --- | --- | --- |
|  |  | **1** | **2** | **3** |  |
| 0.5 | 2.00 | 45.40 | 45.80 | 44.84 | 45.34 ± 0.68 |
| 1 | 1.00 | 23.10 | 22.40 | 23.42 | 22.96 ± 0.72 |
| 3 | 0.33 | 10.64 | 7.33 | 8.06 | 8.68 ± 1.74 |
| 5 | 0.20 | 7.29 | 5.44 | 6.53 | 6.42 ± 0.93 |
| 7 | 0.14 | 5.62 | 4.36 | 5.54 | 5.18 ± 0.70 |

0.05 mM FCLYELAR

| **HHL (mM)** | **1/S** | **1/V** | | | **Average** |
| --- | --- | --- | --- | --- | --- |
|  |  | **1** | **2** | **3** |  |
| 0.5 | 2.00 | 53.09 | 56.49 | 54.20 | 54.59 ± 1.73 |
| 1 | 1.00 | 28.06 | 31.55 | 35.71 | 31.77 ± 5.41 |
| 3 | 0.33 | 15.62 | 16.78 | 17.03 | 16.48 ± 0.75 |
| 5 | 0.20 | 13.85 | 13.82 | 13.77 | 13.81 ± 0.05 |
| 7 | 0.14 | 9.19 | 14.11 | 13.08 | 12.12 ± 2.59 |

0.1 mM FCLYELAR

| **HHL (mM)** | **1/S** | **1/V** | | | **Average** |
| --- | --- | --- | --- | --- | --- |
|  |  | **1** | **2** | **3** |  |
| 0.5 | 2.00 | 62.5 | 69.52 | 60.24 | 64.09 ± 4.84 |
| 1 | 1.00 | 41.32 | 42.61 | 41.78 | 41.91 ± 0.65 |
| 3 | 0.33 | 26.12 | 26.59 | 25.77 | 26.16 ± 0.58 |
| 5 | 0.20 | 22.67 | 21.46 | 22.08 | 22.07 ± 0.86 |
| 7 | 0.14 | 19.45 | 22.03 | 20.12 | 20.53 ± 1.34 |

0.2 mM FCLYELAR

| **HHL (mM)** | **1/S** | **1/V** | | | **Average** |
| --- | --- | --- | --- | --- | --- |
|  |  | **1** | **2** | **3** |  |
| 0.5 | 2.00 | 68.97 | 68.03 | 81.30 | 72.76 ± 7.40 |
| 1 | 1.00 | 52.08 | 50.38 | 52.82 | 51.76 ± 1.25 |
| 3 | 0.33 | 34.26 | 33.61 | 34.80 | 34.22 ± 0.84 |
| 5 | 0.20 | 29.85 | 30.09 | 30.91 | 30.281 ± 0.56 |
| 7 | 0.14 | 28.52 | 29.59 | 28.59 | 28.901 ± 0.59 |
