# Supplementary material for: A novel angiotensin I-converting enzyme inhibitory peptide derived from the trypsin hydrolysates of salmon bone proteins
Source: PLoS One. 2021 Sep 2;16(9):e0256595. doi: 10.1371/journal.pone.0256595 (PMC8412326; doi:10.1371/journal.pone.0256595)
Supplement: Attachment — Submitted filename: Response to Reviewers .docx [file pone.0256595.s008.docx]

**List of changes or point rebuttals**

We would like to thank the reviewers for their careful and thorough reading of this manuscript and for their thoughtful comments and constructive suggestions, which helped to improve the quality of this manuscript. Our response follows (the reviewer’s comments are in yellow highlights).

**Journal requirements:**

When submitting your revision, we need you to address these additional requirements.

1. Please ensure that your manuscript meets PLOS ONE's style requirements, including those for file naming. The PLOS ONE style templates can be found at

https://journals.plos.org/plosone/s/file?id=wjVg/PLOSOne_formatting_sample_main_body.pdf and

https://journals.plos.org/plosone/s/file?id=ba62/PLOSOne_formatting_sample_title_authors_affiliations.pdf

- The suggested correction has been made.

2. In your Data Availability statement, you have not specified where the minimal data set underlying the results described in your manuscript can be found. PLOS defines a study's minimal data set as the underlying data used to reach the conclusions drawn in the manuscript and any additional data required to replicate the reported study findings in their entirety. All PLOS journals require that the minimal data set be made fully available. For more information about our data policy, please see http://journals.plos.org/plosone/s/data-availability.

Upon re-submitting your revised manuscript, please upload your study’s minimal underlying data set as either Supporting Information files or to a stable, public repository and include the relevant URLs, DOIs, or accession numbers within your revised cover letter. For a list of acceptable repositories, please see http://journals.plos.org/plosone/s/data-availability#loc-recommended-repositories. Any potentially identifying patient information must be fully anonymized.

Important: If there are ethical or legal restrictions to sharing your data publicly, please explain these restrictions in detail. Please see our guidelines for more information on what we consider unacceptable restrictions to publicly sharing data: http://journals.plos.org/plosone/s/data-availability#loc-unacceptable-data-access-restrictions. Note that it is not acceptable for the authors to be the sole named individuals responsible for ensuring data access.

We will update your Data Availability statement to reflect the information you provide in your cover letter.

- The suggested correction has been made.

3. Thank you for stating the following in the Acknowledgments Section of your manuscript:

"Furthermore, the authors would also like to make clear their
12 gratitude for the financial support provided by the Thailand Science Research and
13 Innovation (TSRI) Fund (CU_FRB640001_01_61_1), and the Center of Excellence on
14 Medical Biotechnology (CEMB), S&T Postgraduate Education and Research
15 Development Office (PERDO), Office of Higher Education Commission (OHEC),
16 Thailand (SN-63-009-01). The assistance provided by the aforementioned bodies has made
17 this research possible."

We note that you have provided funding information that is not currently declared in your Funding Statement. However, funding information should not appear in the Acknowledgments section or other areas of your manuscript. We will only publish funding information present in the Funding Statement section of the online submission form.

Please remove any funding-related text from the manuscript and let us know how you would like to update your Funding Statement. Currently, your Funding Statement reads as follows:

 "The funders had no role in study design, data collection and analysis, decision to publish, or preparation of the manuscript."

Please include your amended statements within your cover letter; we will change the online submission form on your behalf.

- The suggested correction has been made.

4. Please upload a copy of Figure 8, to which you refer in your text on page 32. If the figure is no longer to be included as part of the submission please remove all reference to it within the text.

- The suggested correction has been made.

**Reviewers' comments:**

**Reviewer's Responses to Questions**

**Comments to the Author**

1. Is the manuscript technically sound, and do the data support the conclusions?

The manuscript must describe a technically sound piece of scientific research with data that supports the conclusions. Experiments must have been conducted rigorously, with appropriate controls, replication, and sample sizes. The conclusions must be drawn appropriately based on the data presented.

Reviewer #1: Yes

Reviewer #2: Yes

2. Has the statistical analysis been performed appropriately and rigorously?

Reviewer #1: Yes

Reviewer #2: Yes

3. Have the authors made all data underlying the findings in their manuscript fully available?

The PLOS Data policy requires authors to make all data underlying the findings described in their manuscript fully available without restriction, with rare exception (please refer to the Data Availability Statement in the manuscript PDF file). The data should be provided as part of the manuscript or its supporting information, or deposited to a public repository. For example, in addition to summary statistics, the data points behind means, medians and variance measures should be available. If there are restrictions on publicly sharing data—e.g. participant privacy or use of data from a third party—those must be specified.

Reviewer #1: Yes

Reviewer #2: Yes

4. Is the manuscript presented in an intelligible fashion and written in standard English?

PLOS ONE does not copyedit accepted manuscripts, so the language in submitted articles must be clear, correct, and unambiguous. Any typographical or grammatical errors should be corrected at revision, so please note any specific errors here.

Reviewer #1: Yes

Reviewer #2: No

5. Review Comments to the Author

Please use the space provided to explain your answers to the questions above. You may also include additional comments for the author, including concerns about dual publication, research ethics, or publication ethics. (Please upload your review as an attachment if it exceeds 20,000 characters)

**Reviewer #1:** Authors have presented a process of enzymatic hydrolysis of salmon bone with trypsin for production of ACE inhibitory peptides.

-Authors should correct some of the mistakes in the English of the manuscript.

The current version has been carefully revised to improve the grammar and readability by Chulalongkorn University Language Institute.

-Authors should discuss the similar studies (e.g., Fu-Yuan Cheng et al., 2009; Fatemeh Mahmoodani et al., 2014; Isabel Rodríguez Amado et al., 2014, etc.) on bone hydrolysis for peptide production, and difference from the present findings should be emphasized.

In this work, similar studies such as the work by Rasli H. and Sarbon N., 2018; Chen J., et al., 2009.; Zhu Z., et al., 2010 have been discussion in the optimization of salmon bone hydrolysis section.

- Conclusion should be more concise.

The current version has been modified.

**Reviewer #2:** Journal: PLOS ONE

Manuscript ID PONE-D-21-03572

Title: A novel angiotensin I-converting enzyme inhibitory peptide derived from the trypsin hydrolysates of salmon bone proteins

General Comments

Thanakrit, et al., extracted a novel angiotensin I-converting enzyme inhibitory peptide from the trypsin hydrolysates of salmon bone proteins. The objectives of manuscript are clear but needs extensive details on its background of the research and goal. As well, some points in the results and discussion part are incomprehensible and require an in-depth explanation.

Authors should answer following questions before publication

1. The manuscript title not providing informative details on the study carried out.

- The manuscript title is appropriate for this article and representative of the content. It also enables readers outside the subject to understand the purpose.

2. English polishing is required before publishing this manuscript.

- The current version has been carefully revised to improve the grammar and readability by Chulalongkorn University Language Institute.

3. Introduction is too vague and descriptive; it must be concise and informative considering aims and objectives of the study.

- The current version has been modified (in yellow highlights).

4. For proximate analysis provide AOAC updated methods from 2000 onwards.

- The AOAC methods were updated in 2005.

5. In every section the authors provided too much descriptive information on each study point, they should provide specific and concrete details on each parameter studied.

-	The current version has been modified

6. Some data must provide as supplementary files.

- The suggested correction has been made.
